# Gut Commensal Fungi Protect Against Acetaminophen-Induced Hepatotoxicity by Reducing *Cyp2a5* Expression in Mice

**DOI:** 10.3389/fmicb.2022.944416

**Published:** 2022-07-12

**Authors:** Zhuoen He, Yunong Zeng, Shuyu Li, Lizhen Lin, Ruisi Zhou, Fangzhao Wang, Wenjiao Yang, Yuhao Wu, Junhao Yang, Ali Chen, Zhang Wang, Hong Yang, Xiaoshan Zhao, Wei Xiao, Lei Li, Shenhai Gong

**Affiliations:** ^1^Department of Critical Care Medicine, The Third Affiliated Hospital of Southern Medical University, Guangzhou, China; ^2^School of Traditional Chinese Medicine, Southern Medical University, Guangzhou, China; ^3^Department of Simulation Center, Zhujiang Hospital of Southern Medical University, Guangzhou, China; ^4^School of Life Science, South China Normal University, Guangzhou, China; ^5^School of Chemistry and Chemical Engineering, Guangdong Pharmaceutical University, Guangzhou, China; ^6^Key Laboratory of Glucolipid Metabolic Disorder, Ministry of Education, Guangdong Pharmaceutical University, Guangzhou, China; ^7^Department of Respiratory and Critical Care Medicine, Affiliated Dongguan Hospital, Southern Medical University, Dongguan, China

**Keywords:** acetaminophen, gut fungi, *Cyp2a5*, acute liver injury, inflammation, oxidative stress

## Abstract

**Background and Aims:**

Drug-induced liver injury (DILI) is a common cause of acute liver failure and represents a significant global public health problem. When discussing the gut-liver axis, although a great deal of research has focused on the role of gut microbiota in regulating the progression of DILI, the gut commensal fungal component has not yet been functionally identified.

**Methods:**

Mice were pretreated with fluconazole (FC) to deplete the gut commensal fungi and were then subject to acetaminophen (APAP) gavage. In addition, transcriptome sequencing was performed to identify differentially expressed genes (DEGs) between control and fluconazole-pretreated groups of the mice challenged with APAP.

**Results:**

Gut commensal fungi ablation through fluconazole pretreatment predisposed mice to APAP-induced hepatotoxicity, characterized by elevated serum liver enzyme levels and more severe centrilobular necrosis, which appears to be caused by robust inflammation and oxidative stress. The 16S rDNA sequencing results indicated that *Akkermansia muciniphila* abundance had significantly decreased in gut fungi-depleted mice, whereas increased abundance of *Helicobacter rodentium* was observed. The gene interaction network between DEGs identified by the transcriptome sequencing highlighted a significant enrichment of *Cyp2a5* in the liver of APAP-treated mice that were preadministrated with fluconazole. Pharmacological inhibition of *Cyp2a5* by 8-methoxypsoralen (8-MOP) could significantly attenuate hepatic inflammation and oxidative stress in mice, thereby conferring resistance to acute liver injury caused by APAP administration.

**Conclusion:**

Our data highlighted the significance of gut commensal fungi in hepatic inflammation and oxidative stress of APAP mice, shedding light on promising therapeutic strategies targeting *Cyp2a5* for DILI treatment.

## Introduction

Drug-induced liver injury (DILI) is becoming an increasingly severe public health problem worldwide as it is one of the leading causes of acute liver failure (ALF; Bernal and Wendon, 2013). A large body of evidence has revealed that acetaminophen (APAP) is extensively used to treat pain and fever, although hepatotoxicity caused by an APAP overdose is the major cause of DILI in many European and North American countries (Larson et al., 2005; Bernal et al., 2013; Stravitz and Lee, 2019). APAP-induced ALF is a progressive disease that is characterized by extensive hepatocellular necrosis and has unacceptably high levels of mortality (Larson et al., 2005; Fernandez-Checa et al., 2021). Although substantial efforts have been made to improve medical management of the condition, APAP-induced liver failure nevertheless causes more than 500 deaths annually in the US (Li et al., 2020). The pathogenesis of APAP hepatotoxicity is linked to the intracellular depletion of hepatic reduced glutathione (GSH), with the consequence of predisposing hepatocytes to mitochondrial reactive oxygen species (ROS; Yuan and Kaplowitz, 2013). A normal dose of APAP can be mostly metabolized in the liver by glucuronidation and sulfation into non-toxic substances, thereby promoting the excretion of APAP *via* the kidneys (Watkins and Seeff, 2006). However, APAP is metabolized to a large number of N-acetyl-p-benzoquinoneimine (NAPQI) through cytochrome P450 enzymes when both glucuronidation and sulfation pathways become saturated following an APAP overdose (Krenkel et al., 2014). After hepatic GSH exhaustion, the excessive NAPQI bonds covalently with hepatocellular macromolecules to form APAP-protein adducts, which leads to hepatocyte necrosis or ferroptosis through mitochondrial dysfunction (Jaeschke et al., 2019; Shojaie et al., 2020).

The gut microbiota is an enormous microscopic community consisting of bacteria, fungi, viruses, and microeukaryotes (Norman et al., 2014). During the dynamic development of the gut microbiome in early life, bacteria-fungi crosstalk is important in maintaining microecological homeostasis and can define the trajectory of the health of the host (Iliev and Leonardi, 2017; Schei et al., 2017). In recent years, the ecological balance in the gut has been shown to regulate the pathogenesis of liver disease *via* the gut-liver axis (Albillos et al., 2020). Prior studies from our laboratory have revealed that the oscillation of gut microbiota mediated the diurnal variation of acetaminophen-induced acute liver injury through the generation of the gut microbial metabolite 1-phenyl-1,2-propanedione (PPD) in mice (Gong et al., 2018). With the increased understanding of intestinal microecology, research is gradually focusing on the potential of the gut fungal component in treating liver disease. For example, the yeast *Saccharomyces boulardii* seems to hinder the growth of pathogenic bacteria, revealing the potential therapeutic implications of certain fungi against infectious disease (Chen et al., 2020). In addition, gut commensal fungal dysbiosis induced by fluconazole (FC) could aggravate allergic airway disease in a house dust mite challenge mode by increasing the infiltration of gut-resident mononuclear phagocytes (MNPs) that express the fractalkine receptor CX3CR1 (Li et al., 2018). However, exactly what role the gut commensal fungi play in preventing the development and progression of drug-induced ALF remains unknown. In the present study, we revealed that gut commensal fungi ablation by fluconazole resulted in hepatic *Cyp2a5* overexpression, thereby increasing susceptibility to APAP hepatotoxicity due to enhanced inflammatory responses and oxidative stress.

## Materials and Methods

### Animal Models

A number of 6–8-week-old male specific-pathogen-free C57BL/6 J mice were housed in standard laboratory conditions under a cycle of 12 h light/12 h dark at room temperature, with food and water *ad libitum*.

The mice were initially treated with fluconazole dissolved in distilled drinking water at a concentration of 0.5 g/l for 14 days (with the solution replaced every 2 days) in order to deplete the gut commensal fungi (Iliev et al., 2012). The mice in the control group were given distilled water. After fluconazole pretreatment, mice were given a single oral dose of 300 mg/kg APAP dissolved in phosphate-buffered saline (PBS) before being sacrificed for tissues collection 24 h later. In addition, mice were treated with a dose of 20 mg/kg 8-methoxypsoralen (8-MOP) dissolved in corn oil to investigate the role of *Cyp2a5* expression toward inhibiting inflammatory responses and oxidative stress in hepatotoxicity caused by APAP.

All experimental procedures were performed in accordance with the National Institutes of Health guidelines and were approved by the local Animal Care and Use Committee of Southern Medical University, Guangzhou, China.

### Biochemical Analysis

The levels of alanine transaminase (ALT) and aspartate transaminase (AST) in the serum were quantitated using commercial assay kits (Jiancheng, Nanjing, China) according to the manufacturer’s instructions. Malondialdehyde (MDA), superoxide dismutase (SOD), and glutathione (GSH) activity in the liver tissues were determined using a corresponding commercial assay kit (Jiancheng, Nanjing, China). TNF-α, IL-6, and MCP-1 concentrations in the plasma were measured by the ELISA assay kits (Neobioscience, Shenzhen, China), and the serum MCP-3 level was detected with an ELISA assay kit (CUSABIO, Wuhan, China).

### Histopathological Analysis

Liver tissues were fixed in 4% paraformaldehyde (PFA) for 24 h at room temperature, before being embedded in paraffin and sectioned. Hematoxylin and eosin (HE) staining were performed to assess pathological changes in the liver. At least 6–8 randomly selected fields per sample were used to calculate the area of liver necrosis over the whole field using ImageJ software (National Institute of Health).

### Immunohistochemical Staining

The paraffin-embedded slides were dewaxed, rehydrated, and incubated in 3% H_2_O_2_ for 20 min and calf serum for 15 min, respectively. Next, the slides were incubated with anti-CD11b antibody (Servicebio, Wuhan, China) at 4°C overnight, before being incubated with horseradish peroxidase (HRP)-labeled secondary antibody (Gene Tech, Shanghai, China). After washing with PBS, the slides were stained with DAB and hematoxylin, respectively. Finally, eight fields per slide were randomly photographed in order to assess and quantify CD11b-positive cells using ImageJ software.

### Fluorescence Staining

The paraffin-embedded liver sections were subjected to terminal-deoxynucleotidyl transferase-mediated nick end labeling (TUNEL) staining using a commercial assay kit (KeyGEN BioTECH, Nanjing, China) according to the manufacturer’s instructions. At least 6–7 fields per slide were randomly selected in order to determine the number of TUNEL-positive cells.

To detect the hepatic ROS levels, the frozen liver sections were incubated with dihydroethidium (DHE, Thermo Scientific, MA, United States) at a final concentration of 2 μM at 37°C for 30 min. Between four and eight fields per slide were then selected at random and the mean fluorescence intensity (MFI) was analyzed using ImageJ software.

### Microbial Analysis

Feces from mice were collected on day 14 after fluconazole treatment and were immediately stored at-80°C. The fecal samples were mashed in PBS containing 0.5% Tween20 solution and were then subjected to repeated freezing at-80°C for 10 min and thawing at 60°C for 5 min. The next steps involved extracting and purifying the DNA using the phenol-chloroform isoamyl alcohol method and a commercial reagent (Solarbio, Beijing, China). Next, the concentration and purity of total DNA were determined by using a NanoDrop spectrophotometer (Thermo Scientific, MA, United States). The hypervariable region 4 (V4) of the bacterial 16S rRNA was amplified by a polymerase chain reaction (PCR) using primers V4-F (5′-GTGTGYCAGCMGCCGCGGTAA-3′) and V4-R (5′-CCG GACTACNVGGGTWTCTAAT-3′). DNA sequencing was performed on an Illumina Hiseq PE250, and the raw data of 16S rRNA gene sequencing were analyzed with the QIIME2 platform (v2020.2).

### Quantitative Real-Time PCR Analysis

RNA was extracted from the liver tissues using TRIzol reagent (Thermo Scientific, MA, United States) and separated by chloroform. cDNA was obtained by reverse transcription with ReverTra Ace qPCR RT Kit (Toyobo, Shanghai, China). Quantitative real-time PCR (qRT-PCR) analysis were performed on a 7,500 Real-Time PCR System (Applied Biosystems, CA, United States) and 18S ribosomal RNA was used for normalization and quantification of the target gene expression levels using the comparative CT method. All primers for qRT-PCR analysis were listed in Table 1.

**Table 1:** Primers for qPCR.

	Forward primer	Reverse primer
*18S*	AGTCCCTGCCCTTTGTACACA	CGATCCGAGGGCCTCACTA
*16S*	TGATGCACTTGCAGAAAACA	ACCAGAGGAAATTTTCAATAGGC
*Ccl2*	TAAAAACCTGGATCGGAACCAAA	GCATTAGCTTCAGATTTACGGGT
*Ccl3*	TGTACCATGACACTCTGCAAC	CAACGATGAATTGGCGTGGAA
*Ccl7*	CCACATGCTGCTATGTCAAGA	ACACCGACTACTGGTGATCCT
*Ptges*	GGATGCGCTGAAACGTGGA	CAGGAATGAGTACACGAAGCC
*Cbr3*	CATCGGCTTTGCGATCACG	GACCAGCACGTTAAGTCCCC
*Firmicutes*	GGAGYATGTGGTTTAATTCGA	AGCTGACGACAACCATGCAC
*Bacteroidetes*	GGCGACCGGCGCACGGG	GRCCTTCCTCTCAGAACCC
*A. muciniphila*^a^	CAGCACGTGAAGGTGGGGAC	CCTTGCGGTTGGCTTCAGAT
*H. rodentium*^b^	GTGGAGTGCTAGCTTGCTAGAA	ACCGTAGCATAGCTGATCTA
*FungiQuant*	GGRAAACTCACCAGGTCCAG	GSWCTATCCCCAKCACGA
*Cyp2a5*	TGGTCCTGTATTCACCATCTACC	ACTACGCCATAGCCTTTGAAAA

a *Akkermansia muciniphila.*

b *Helicobacter rodentium.*

### Western Blot

Liver tissues were homogenized in RIPA lysis buffer (Beyotime, Shanghai, China) on ice to prepare for total protein extraction. Total protein extractions were denatured with SDS loading buffer by heating at 95°C for 5 min. After blocking with 5% non-fat milk in tris-buffered saline tween (TBST), the membranes were incubated separately with total JNK (Cell Signaling Technology, MA, United States), p-JNK (Cell Signaling Technology, MA, United States), total p65 (Cell Signaling Technology, MA, United States), p-p65 (Cell Signaling Technology, MA, United States), total p38 (Cell Signaling Technology, MA, United States), p-p38 (Cell Signaling Technology, MA, United States), total ERK (Cell Signaling Technology, MA, United States), and p-ERK (Cell Signaling Technology, MA, United States) antibodies overnight at 4°C. Finally, the protein bands were incubated with secondary antibodies (Proteintech, Wuhan, China) for 1 h at room temperature and were then visualized using an enhanced chemiluminescence (ECL) detection kit (Vazyme, Wuhan, China).

### Transcriptomic Sequencing Analysis

To conduct the transcriptomic sequencing analysis, the total RNA from the livers of the APAP-treated mice with or without fluconazole pretreatment was extracted using TRIzol reagent (Thermo Scientific, MA, United States). Purified total RNA was performed to construct libraries, followed by subjection to sequence through Illumina NovaSeq 6,000 (Novogene Co., Ltd., Beijing, China). Statistical analysis was performed with the DESeq2 R package. The genes with |log2 (fold change)| > 1 and adjusted *p* < 0.05 were identified as differentially expressed genes (DEGs).

### Data Availability Statement

The transcriptome and 16S rDNA data have been uploaded to the China Nucleotide Sequence Archive (CNSA, https://db.cngb.org/cnsa/) under accession codes CNP0002810 and CNP0002825, respectively.

### Statistical Analysis

All data were expressed as mean ± standard error of the mean (SEM) and were evaluated by an unpaired two-tailed Student’s *t*-test. A value of *p* lower than 0.05 was considered significant (^*^p < 0.05).

## Results

### Gut Commensal Fungi Protect Mice Against APAP-Induced Acute Liver Failure

**Figure 1:**
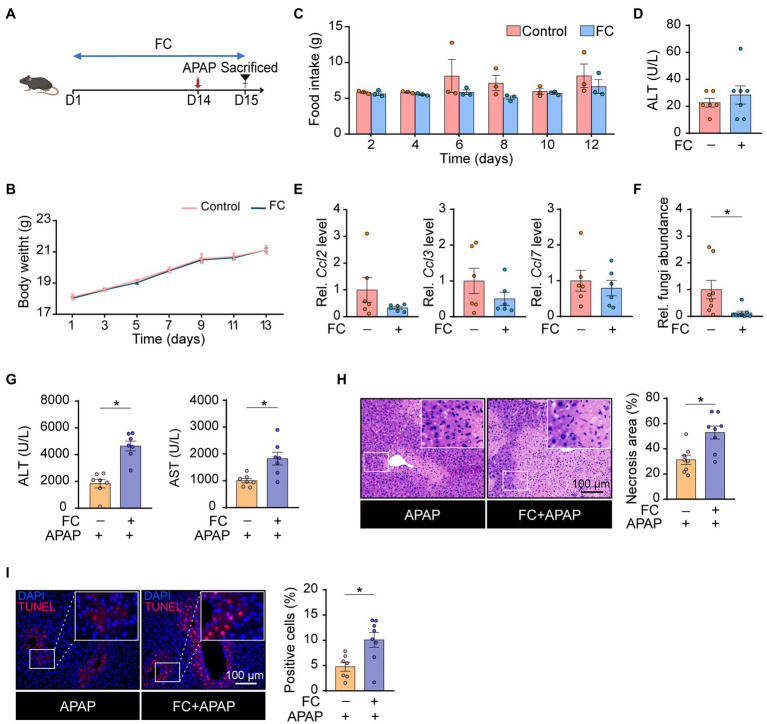
Gut commensal fungi protect mice against APAP-induced acute liver failure. **(A)** Study design. C57BL/6 mice were pretreated with fluconazole for 14 days to deplete the gut commensal fungi, followed by subjection to APAP administration for 24 h. Tissues were collected after mice were euthanized. **(B)** Body weight change in the control and fluconazole-only group. **(C)** Food intake of the control and fluconazole-only group. **(D)** Effect of fluconazole administration alone on serum ALT levels. **(E)** Relative mRNA levels of *Ccl2, Ccl3,* and *Ccl7* in the liver of control and fluconazole-only group were detected by qRT-PCR. **(F)** Relative abundance of fungi in the gut of the control and fluconazole-only group. **(G)** Plasma ALT and AST levels in APAP-treated mice with or without preadministration of fluconazole. **(H)** Representative H&E staining images and quantification of necrotic areas in the livers of APAP-treated mice with or without preadministration of fluconazole. **(I)** TUNEL staining of the liver from APAP-treated mice with or without preadministration of fluconazole and quantification of dead cells. ^*^p < 0.05. Data were expressed as mean ± SEM and were evaluated by a two-tailed unpaired Student’s *t*-test. *n* = 3–8. Scale bar = 100 μm. FC, fluconazole; APAP, acetaminophen; ALT, alanine aminotransferase; AST, aspartate aminotransferase; Rel, Relative; H&E, hematoxylin and eosin; and TUNEL, terminal-deoxynucleotidyl transferase-mediated nick end labeling.

To explore the role of gut commensal fungi in APAP-induced hepatotoxicity, the mice were orally pretreated with fluconazole (FC) for 14 days to deplete the gut commensal fungi, followed by subjection to APAP administration (Figure 1A). First, we determined whether the gut commensal fungi depletion through fluconazole administration affected energy intake. As shown in Figures 1B,C, we found that food consumption and body weight were not altered by the presence of fluconazole in mice. Serum biochemistry results showed that ALT levels were comparable in control and fluconazole-treated mice (Figure 1D), which was further confirmed by the hepatic expression of inflammatory chemokine including *Ccl2*, *Ccl3* and *Ccl7* (Figure 1E). The above data suggested that the dose of fluconazole used in our study did not directly cause hepatotoxicity. Consistent with previous study (Qiu et al., 2015), the qRT-PCR result showed that the fluconazole-treated mice had a lesser abundance of gut fungi in comparison with the control group (Figure 1F). Interestingly, gut fungi ablation caused by the fluconazole pretreatment resulted in a significant increase in plasma ALT and AST levels in response to APAP administration (Figure 1G). Histopathological examination displayed that gut commensal fungi depletion significantly elevated the area of necrosis in the liver tissues of APAP-treated mice (Figure 1H). This result was further supported by the TUNEL staining, which measured the level of cell death including necrosis, apoptosis and necroptosis (Figure 1I; Grasl-Kraupp et al., 1995). All of the above-mentioned results indicated that gut commensal fungi depletion predisposed mice to APAP-induced hepatotoxicity.

### Gut Commensal Fungi Depletion Alters Bacterial Community Structure in Mice

**Figure 2:**
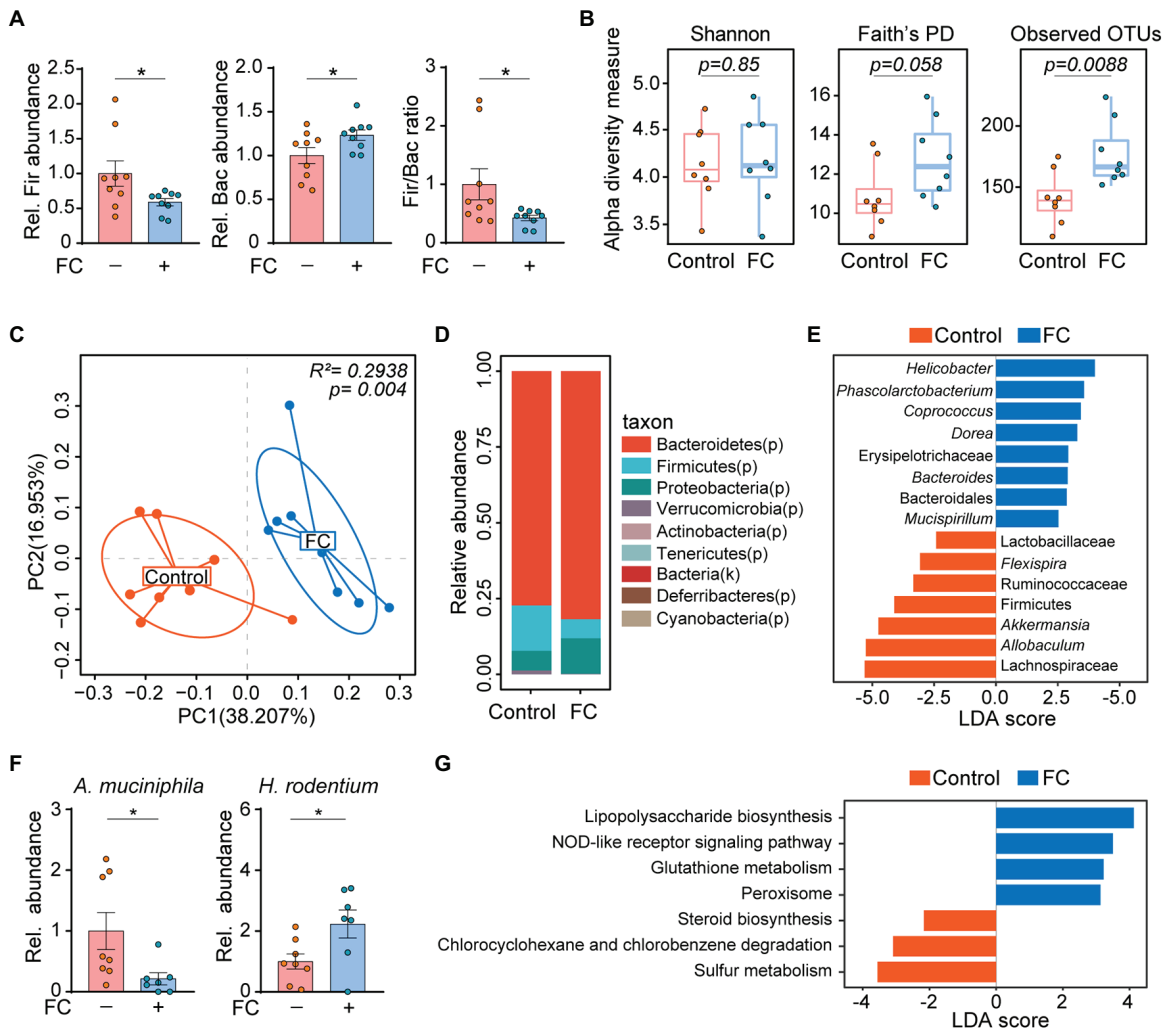
Gut commensal fungi depletion alters bacterial community structure in mice. **(A)** C57BL/6 mice were treated with fluconazole to deplete the gut commensal fungi. Feces were collected after 14 days of fluconazole treatment. Relative abundance of *Firmicutes* and *Bacteroidetes* as well as the ratio of *Firmicutes*/*Bacteroidetes* in the feces of control and fluconazole-only group. **(B)** Alpha diversity based on Shannon Index, Faith’s PD, and Observed OTUs of gut microbiota in the control and fluconazole-only group. These data were evaluated by Wilcoxon rank-sum test. **(C)** Principal coordinate analysis (PCoA) using Bray–Curtis distance of microbial composition in control and fluconazole-treated mice. These data were evaluated by the Adonis test. **(D)** Relative abundance of gut microbiota at phylum level from control and fluconazole-treated mice. **(E)** The differences in specific microbiota taxa between the control and fluconazole-treated mice were identified by linear discriminant analysis effect size (LEfSe) analysis. **(F)** Relative abundance of *Akkermansia muciniphila* and *Helicobacter rodentium* in control and fluconazole-treated mice. **(G)** Predicated functional KEGG pathways were inferred from OTUs by PICRUSt analysis in control and fluconazole-treated mice. ^*^p < 0.05. All bar graph data were expressed as mean ± SEM and were evaluated by a two-tailed unpaired Student’s *t*-test. *n* = 7–9. Rel, Relative; FC, fluconazole; Fir, *Firmicutes*; Bac, *Bacteroidetes*; *A. muciniphila*, *Akkermansia muciniphila*; *H. rodentium*, *Helicobacter rodentium*; LEfSe, linear discriminant analysis effect size; and LDA, linear discriminant analysis.

A previous study had shown that the interactions between gut commensal fungi and bacteria are important for maintaining intestinal health and improving disease resistance (Sam et al., 2017). For this reason, we next performed 16S rDNA sequencing to determine the alterations in gut bacterial community structure in response to fluconazole treatment. The qRT-PCR result indicated a significant decrease in the *Firmicutes*/*Bacteroidetes* ratio, reflecting the composition of gut microbes in mice subjected to fluconazole treatment for 14 days (Figure 2A). There were significant changes in the alpha diversity of gut bacteria as shown by Faith’s Phylogenetic Distance (PD) and Observed OTUs, although not in the Shannon diversity index (Figure 2B). Consistently, significant intergroup differences among control and fluconazole-treated mice were assessed using the Bray-Curtis metric distance according to the principal coordinates analysis (Figure 2C). Moreover, gut fungi ablation through fluconazole treatment induced a distinct clustering of microbiota composition at the phylum level in mice (Figure 2D).

Specially, the linear discriminant analysis (LDA) effect size (LEfSe) statistical analysis indicated that mice subjected to fluconazole treatment were enriched for members of genus *Helicobacter* and *Dorea*, whereas genus *Akkermansia* and *Allobaculum* were enriched in control group (Figure 2E). To validate these results, we performed qRT-PCR to determine the changes in gut microbiota in response to fluconazole treatment and found that the abundance of *Akkermansia muciniphila* (*A. muciniphila*) was decreased, however, *Helicobacter rodentium* (*H. rodentium*) abundance exhibited an opposite trend in fluconazole-treated mice compared with the control group (Figure 2F). PICRUSt analysis on the OTU derived from the 16S rDNA sequence was also conducted to predict potential metabolic functions of gut microbiota in fluconazole-treated mice. In company with increased abundance of *Helicobacter*, which is defined as pathogens associated with generation of lipopolysaccharides (Hynes et al., 2004), the genomic abundance of some pathways, which included lipopolysaccharide biosynthesis and NOD-like receptor signaling pathway were significantly enhanced in fluconazole-treated mice compared with the control group (Figure 2G). In contrast, we also found that some pathways including sulfur metabolism and steroid biosynthesis were enriched in the control group (Figure 2G). All of the above data demonstrated that the crosstalk of bacteria and fungi is important in maintaining microecological homeostasis.

### Gut Commensal Fungi Ablation Promotes the Inflammatory Response Associated With Aberrant Arachidonic Acid Metabolism in APAP-Treated Mice

**Figure 3:**
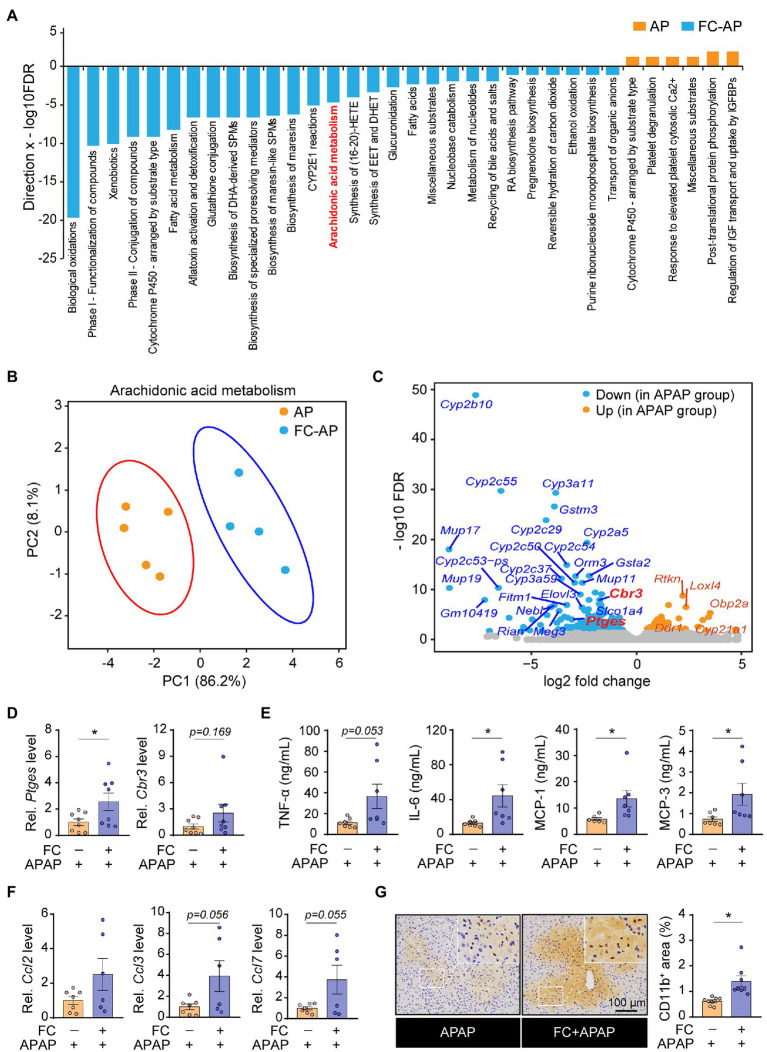
Gut commensal fungi ablation promotes the inflammatory response associated with aberrant arachidonic acid metabolism in APAP-treated mice. **(A)** Hypergeometric analysis showing pathway enrichment analysis of the liver tissues from APAP-treated mice with or without preadministration of fluconazole. **(B)** The principal component analysis (PCA) of arachidonic acid metabolism is based on transcriptomic analysis in APAP-treated mice with or without preadministration of fluconazole. **(C)** Volcano plot of DEGs in the control group compared with fluconazole-pretreated mice in presence of APAP administration. **(D)** Relative mRNA levels of *Ptges* and *Cbr3* in liver tissues of APAP-treated mice with or without preadministration of fluconazole. **(E)** Quantification of pro-inflammatory cytokines including TNF-α, IL-6, MCP-1 and MCP-3 in the blood from APAP-treated mice with or without preadministration of fluconazole. **(F)** Relative mRNA levels of *Ccl2, Ccl3,* and *Ccl7* in the liver of APAP-treated mice with or without preadministration of fluconazole were detected by qRT-PCR. **(G)** Immunohistochemical staining of CD11b and quantification of positive cells in the liver tissues of APAP-treated mice with or without preadministration of fluconazole. ^*^p < 0.05. All bar graph data were expressed as mean ± SEM and were evaluated by a two-tailed unpaired Student’s *t*-test. *n* = 4–8. Scale bar = 100 μm. APAP, acetaminophen; FC, fluconazole; and Rel, Relative.

To explore how gut commensal fungi protected mice against APAP-induced acute liver injury, we performed transcriptome analysis of the liver tissues collected from the APAP-treated mice subjected to fluconazole preadministration. Notably, the KEGG pathway enrichment analysis of DEGs showed that the pathway of arachidonic acid metabolism was enhanced in fluconazole-pretreated mice compared with the control group in the presence APAP administration (Figure 3A), which was also confirmed by PCA analysis (Figure 3B). Concomitantly, volcano plots displayed that mice subjected to fluconazole pretreatment exhibited an increase in the hepatic mRNA levels of the *Ptges* and *Cbr3* genes responsible for inflammatory mediator prostaglandin E2 biosynthesis and metabolism in the presence of APAP (Figure 3C). The DEGs identified by the transcriptome analysis were also convincingly supported by real-time quantitative PCR analysis (Figure 3D).

Emerging evidence indicated that the aberrant metabolism of arachidonic acid profoundly triggers an inflammatory response in the host, thus resulting in an acceleration of the disease process (Harizi et al., 2008). Therefore, it was reasonable to speculate that gut commensal fungi ablation through fluconazole treatment predisposed mice to hepatotoxicity caused by APAP overdose, which appeared to be due to vigorous proinflammatory cytokine production. Indeed, compared with control mice, fluconazole pretreatment resulted in a larger increase in the concentrations of cytokine and chemokine including TNF-α, IL-6, MCP-1 and MCP-3 in the blood of APAP-treated mice (Figure 3E). In addition, we also observed that gut commensal fungi depletion exacerbated hepatic inflammatory responses in response to APAP administration, as indicated by the increase in the mRNA levels of chemokines (Figure 3F). Furthermore, immunohistochemical (IHC) staining displayed that the number of infiltrating CD11b-positive cells, defined as inflammatory cells (Solovjov et al., 2005), increased in the liver tissues of APAP-treated mice who had been pretreated with fluconazole (Figure 3G). In all, the above results suggested that the gut fungi ablation by fluconazole pretreatment have enhanced hepatic inflammation in mice upon APAP exposure.

### Gut Commensal Fungi Ablation Exacerbates Hepatic Oxidative Stress in APAP-Treated Mice

**Figure 4:**
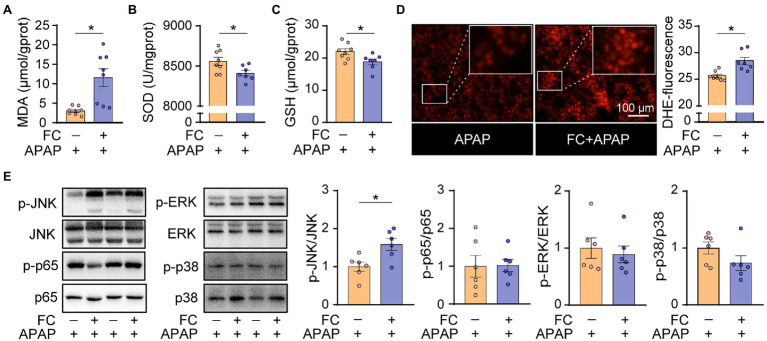
Gut commensal fungi ablation exacerbates hepatic oxidative stress in APAP-treated mice. **(A–C)** C57BL/6 mice were pretreated with fluconazole for 14 days to deplete the gut commensal fungi and were then subjected to APAP administration for 1 h. Hepatic MDA **(A)**, SOD **(B)** and total GSH **(C)** levels in APAP-treated mice with or without preadministration of fluconazole. **(D)** Representative images of DHE immunofluorescence staining and quantification of hepatic ROS production in APAP-treated mice with or without preadministration of fluconazole. **(E)** Western blot was performed with specific antibodies to quantify the phosphorylated JNK, ERK, p38 and p65 in the liver tissues from APAP-treated mice with or without preadministration of fluconazole. ^*^p < 0.05. Data were expressed as mean ± SEM and were evaluated by a two-tailed unpaired Student’s *t*-test. *n* = 6–8. Scale bar = 100 μm. APAP, acetaminophen; FC, fluconazole; MDA, malonaldehyde; SOD, superoxide dismutase; GSH, glutathione; and DHE, dihydroethidium.

A previous study demonstrated that the depletion of hepatic reduced glutathione and the subsequent oxidative stress is involved in the pathogenesis of APAP-induced acute liver injury (Goldring et al., 2004). In order to further understand the underlying mechanism by which gut fungi ablation enhanced hepatotoxicity induced by APAP in mice, we thus determined the level of oxidative stress in the liver from APAP-treated mice subjected to fluconazole pretreatment. We first found that hepatic malondialdehyde (MDA) levels in APAP-treated mice subjected to fluconazole preadministration were significantly higher than in the APAP-only group (Figure 4A). Correspondingly, the markers of antioxidant capacity, SOD activity and GSH levels were significantly repressed in the livers of APAP-treated mice subjected to fluconazole preadministration (Figures 4B,C). In addition, DHE staining showed that fluconazole preadministration significantly increased ROS accumulation in the liver of APAP-treated mice (Figure 4D). We next focused on the down signaling pathway which was affected by fluconazole preadministration in APAP-treated mice. As shown in Figure 4E, the phosphorylation level of JNK was significantly increased in the livers of APAP-treated mice subjected to fluconazole preadministration compared with the APAP-only group, whereas other MAPK and NF-κB signaling pathways remained comparable in the two groups. All of these data indicated that fluconazole-induced oxidative stress and sustained JNK activation predisposed mice to APAP-induced hepatotoxicity.

### Gut Commensal Fungi Ablation Enhances the Susceptibility to APAP-Induced Hepatotoxicity by Upregulation of Hepatic *Cyp2a5* Level

**Figure 5:**
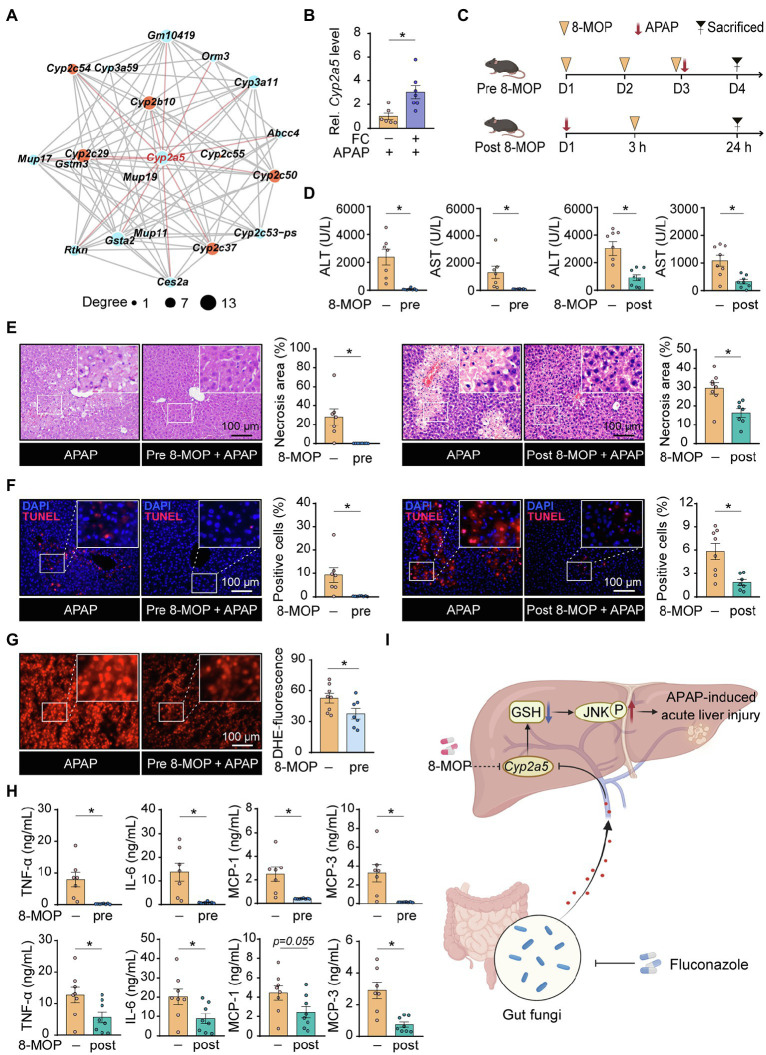
Gut commensal fungi ablation enhances the susceptibility to APAP-induced hepatotoxicity by upregulation of hepatic *Cyp2a5* level. **(A)** The interaction network of the top twenty DEGs in the control group compared with fluconazole-pretreated mice in presence of APAP administration. C57BL/6 mice were pretreated with fluconazole for 14 days to deplete the gut commensal fungi, followed by subjection to APAP administration for 24 h. Liver tissues were collected for transcriptome sequencing analysis. **(B)** Relative mRNA levels of *Cyp2a5* in the liver tissues from APAP-treated mice with or without preadministration of fluconazole. **(C)** Study design. Mice were pre- and post-treated with the *Cyp2a5* inhibitor, 8-MOP, in presence of APAP administration. After 24 h of APAP treatment, the mice were sacrificed, and tissues were collected for further analysis. **(D)** Effect of 8-MOP on ALT and AST levels in the blood of APAP-treated mice. **(E)** Representative H&E staining images and quantification of necrotic areas in the liver from APAP-treated mice with or without administration of 8-MOP. **(F)** TUNEL staining of the liver from APAP-treated mice with or without administration of 8-MOP and quantification of dead cells. **(G)** Representative images of DHE immunofluorescence staining and quantification of hepatic ROS production in APAP-treated mice with or without preadministration of 8-MOP. **(H)** Quantification of pro-inflammatory cytokines including TNF-α, IL-6, MCP-1 and MCP-3 in the blood of APAP-treated mice with or without 8-MOP administration. **(I)** Working model: Mice with gut fungi depletion by fluconazole treatment showed more sensitivity to APAP-induced acute liver injury through the promotion of hepatic *Cyp2a5* overexpression (Created with BioRender.com). ^*^p < 0.05. Data were expressed as mean ± SEM and were evaluated by a two-tailed unpaired Student’s *t*-test. *n* = 6–8. Scale bar = 100 μm. APAP, acetaminophen; FC, fluconazole; Rel, Relative; 8-MOP, 8-methoxypsoralen; ALT, alanine aminotransferase; AST, aspartate aminotransferase; H&E, hematoxylin and eosin; TUNEL, terminal-deoxynucleotidyl transferase-mediated nick end labeling; and DHE, dihydroethidium.

To further illustrate the underlying molecular mechanism of fluconazole pretreatment responsible for excessive inflammatory response and oxidative stress in APAP-treated mice, we performed transcriptome analysis and selected the top twenty DEGs ranked by value of *p* for interaction network analysis. Figures 5A,B show that the expression of *Cyp2a5*, identified as the hub gene and highlighted in red, was increased in liver tissues of APAP-treated mice subjected to fluconazole pretreatment. On the basis of these observations, we considered that gut commensal fungi ablation through fluconazole treatment could promote hepatic *Cyp2a5* overexpression, thereby resulting in increasing the susceptibility to APAP-induced hepatotoxicity in mice.

To verify this hypothesis, mice were pre- and post-treated with 8-methoxypsoralen (8-MOP, known as a *Cyp2a5* inhibitor) in the presence of APAP administration, respectively (Figure 5C). Interestingly, pharmacologic inhibition of *Cyp2a5*, by administration of 8-MOP to control mice, fully reversed the hepatotoxicity induced by APAP administration, as indicated by the result of serum biochemistry (Figure 5D). Furthermore, histopathological examination displayed that the percentage of cell death apparently decreased in the liver tissues of APAP-treated mice subjected to 8-MOP treatment compared with the APAP-only group (Figures 5E,F). In line with the above results, pharmacologic inhibition of *Cyp2a5* significantly suppressed the accumulation of hepatic ROS as well as cytokine and chemokine release in APAP-treated mice (Figures 5G,H). Taken together, these discoveries suggested that inhibiting hepatic *Cyp2a5* overexpression may hold promise as a novel pharmacological strategy for treating APAP-induced acute liver injury.

## Discussion

DILI is a common acute liver disease characterized by high mortality and is mainly caused by an overdose of APAP in Western countries (Andrade et al., 2019). The pathogenesis of APAP-induced hepatotoxicity is associated with the persistent depletion of hepatic reduced glutathione (GSH), leading to predispose hepatocytes to sustained oxidative stress-induced necroptosis and ferroptosis (Matsumaru et al., 2003; Yamada et al., 2020). The incidence of DILI was progressively increased worldwide that accompany with the action of abuse drugs (Haque et al., 2016). Therefore, novel targeted strategies for the treatment of drug-induced hepatotoxicity are essential.

Although maintaining the ecological homeostasis in the gut has recently been shown to increase resistance to ALF, the biological functions of gut commensal fungi in regulation of the sustained oxidative stress during acute liver injury induced by APAP overdose remain largely undefined (Chopyk and Grakoui, 2020). Our data clearly revealed that fluconazole pretreatment predisposed mice to accelerated hepatotoxicity induced by APAP. We next explored whether the effects of fluconazole in the progression of APAP-induced liver injury were in gut fungi-dependent manner. It is well known that glutathione biosynthesis is dependent on the uptake of amino acids during food processing and digestion (Minich and Brown, 2019). We first monitored food consumption and body weight periodically until the end of the fluconazole treatment period to exclude the possibility that any difference in energy metabolism between the fluconazole-treated mice and the control group influenced the process of glutathione biosynthesis in the liver. Additionally, we also found that fluconazole treatment alone did not cause liver injury in our study, as confirmed by the serum level of ALT, and the expression of chemokines in control and fluconazole-treated mice, whereas we found that the gut fungi load was significantly reduced in fluconazole-treated mice compared with the control group, a result consistent with previous literature (Li et al., 2018). These above results indicated that gut commensal fungi could protect mice against APAP-induced liver injury, which suggested that maintaining the gut commensal fungi balance could be a promising therapeutic strategy for preventing DILI. Further preclinical and clinical research will be necessary to identify the specific intestinal-resident fungi responsible for protecting against drug-induced hepatotoxicity.

Since commensal bacteria coexist with fungi in the intestine, maintaining microecological homeostasis can increase resistance to disease through the modulation of host immunity (Clemente et al., 2012). This notion is supported by the other observation that antibiotic-mediated depletion of intestinal-resident bacteria may shift the organizational structure of gut commensal fungi, leading to result in an overabundance of *Saccharomycetes* fungi in particular (Jiang et al., 2017). However, less is known about whether fluconazole-induced fungi depletion leads to persistent changes in the composition of gut bacteria. In the present study, we noted that gut commensal fungi ablation through fluconazole treatment could increase the diversity of intestinal bacteria communities, as shown by Faith’s Phylogenetic Distance (PD) and the Observed OTUs, which appears to be due to a strong competition between enteric bacterial and fungal components for commonly available nutrients in a shared environment. In addition, our data showed that the relative ratio of *Firmicutes* and *Bacteroidetes* was decreased in fluconazole-treated mice compared with the control group, indicating the occurrence of a gut microbiota disorder. In particular, we found that gut fungi ablation leads to an overabundance of the enteric bacterial pathogen *H. rodentium*, while the level of probiotic *A. muciniphila* was decreased. Therefore, our study has provided new insight into the significance of enteric bacteria-fungi interactions, although the functions of the bacteria or its derived metabolites modulated by fungal components in the gut need further clarification.

A key finding of the current study was the highlighting of inhibition of hepatic *Cyp2a5* expression as a novel therapeutic strategy for treating drug-induced liver failure. As a member of the CYP2A subfamily, murine *Cyp2a5*, and its human ortholog, *Cyp2a6*, have been identified as important xenobiotic-metabolizing enzymes in the liver (Kim et al., 2005; Kirby et al., 2011). Recent research indicated that exposure to chemicals including ethanol, thioacetamide, and cadmium could promote upregulations of hepatic *Cyp2a5* expression in mice, thereby modulating disease susceptibility by affecting oxidative stress (Lu et al., 2011; Hong et al., 2016). However, the pathogenic role of *Cyp2a5* in regulating acute liver injury induced by APAP overdose remains unclear. In the present study, we found that the expression of *Cyp2a5* was increased in APAP-treated mice subjected to fluconazole pretreatment, suggesting that *Cyp2a5* appears to be a dominant-positive regulator of APAP hepatotoxicity. To verify this hypothesis, we applied a known *Cyp2a5* inhibitor, 8-methoxypsoralen (8-MOP), to APAP-treated mice and found that the pharmacologic inhibition of *Cyp2a5* fully reversed the hepatotoxicity induced by APAP administration.

Given the possibility that the protective role of 8-MOP on APAP-induced hepatotoxicity was mediated through the blocking of APAP metabolic bioactivation, mice were treated with 8-MOP after 3 h of APAP administration, by which time the processes of intrahepatic APAP metabolic is end (Souza et al., 2022). Serum biochemical analysis showed that administering 8-MOP post-treatment also reduced APAP-induced hepatotoxicity in mice, a conclusion which was further confirmed by histopathological examination. The pharmacologic inhibition of *Cyp2a5*, therefore, could play a role in preventing drug-induced liver failure, although the potential therapeutic effects of 8-MOP will require further clinical study.

In summary, our findings identified the protective role of gut commensal fungi against acute APAP-induced liver injury through an attenuated inflammatory response and oxidative stress in mice and have shed light on the significance of downregulating *Cyp2a5* by 8-MOP as a novel pharmacological strategy for treating DILI.

## Data Availability Statement

The datasets presented in this study can be found in online repositories. The names of the repository/repositories and accession number(s) can be found in the article/Supplementary Material.

## Ethics Statement

The animal study was reviewed and approved by the Institutional Animal Care and Use Committee of Southern Medical University, Guangzhou, China.

## Author Contributions

SG, LL, WX, XZ, and HY were responsible for the study design, supervision, and manuscript preparation. ZH, YZ, SL, LL, RZ, FW, WY, and YW were responsible for the experiment. JY, AC, and ZW were responsible for sequencing analysis. All authors contributed to the article and approved the submitted version.

## Funding

This work was financially supported by the National Natural Science Foundation of China (81973804), Shenzhen Science and Technology Program (RCBS20210706092252059), the Key Project of National Natural Science Foundation of China (81830117), and the National Natural Science Foundation of China (82104382).

## Conflict of Interest

The authors declare that the research was conducted in the absence of any commercial or financial relationships that could be construed as a potential conflict of interest.

## Publisher’s Note

All claims expressed in this article are solely those of the authors and do not necessarily represent those of their affiliated organizations, or those of the publisher, the editors and the reviewers. Any product that may be evaluated in this article, or claim that may be made by its manufacturer, is not guaranteed or endorsed by the publisher.

## Abbreviations

DILI: drug-induced liver injury
ALF: acute liver failure
APAP: acetaminophen
GSH: glutathione
ROS: reactive oxygen species
NAPQI: n-acetyl-p-benzoquinoneimine
PPD: 1-phenyl-1,2-propanedione
MNPs: mononuclear phagocytes
FC: fluconazole
PBS: phosphate-buffered saline
8-MOP: 8-methoxypsoralen
ALT: alanine transaminase
AST: aspartate transaminase
MDA: malondialdehyde
SOD: superoxide dismutase
PFA: paraformaldehyde
HE: hematoxylin and eosin
HRP: horseradish peroxidase
TUNEL: terminal-deoxynucleotidyl transferase-mediated nick end labeling
DHE: dihydroethidium
MFI: mean fluorescence intensity
PCR: polymerase chain reaction
qRT-PCR: quantitative real-time polymerase chain reaction
TBST: tris-buffered saline tween
ECL: enhanced chemiluminescence
DEGs: differentially expressed genes
SEM: standard error of the mean
PD: phylogenetic distance
LDA: linear discriminant analysis
LEfSe: linear discriminant analysis effect size
*A. muciniphila*: *Akkermansia muciniphila*
*H. rodentium*: *Helicobacter rodentium*
IHC: immunohistochemical
PCoA: principal coordinate analysis
PCA: principal component analysis
