# Supplementary material for: Gut Commensal Fungi Protect Against Acetaminophen-Induced Hepatotoxicity by Reducing Cyp2a5 Expression in Mice
Source: Front Microbiol. 2022 Jul 12;13:944416. doi: 10.3389/fmicb.2022.944416 (PMC9315200; doi:10.3389/fmicb.2022.944416)
Supplement: Supplementary file 1 [file Image_1.pdf]

Supplementary Material

Supplementary Figure

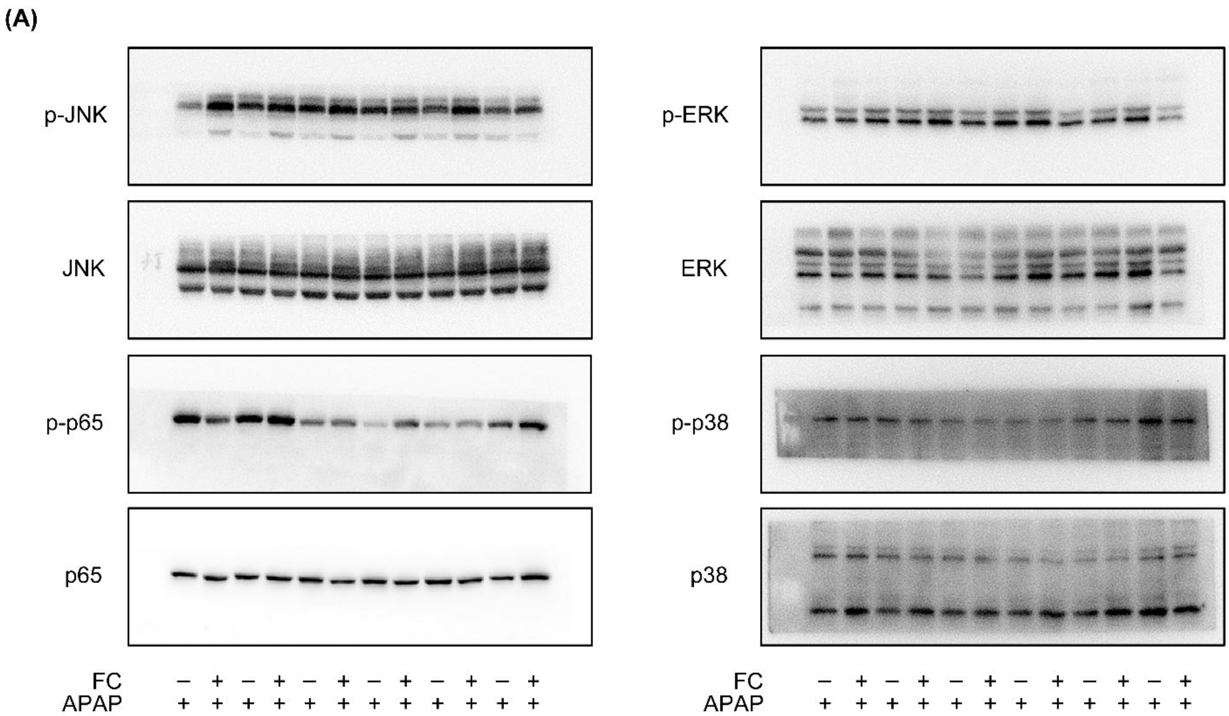

Supplementary Figure 1. The full scan of the western blot in **Figure 4C**.
